# Association of elevated glycosylated hemoglobin A1c with hyperfiltration in a middle-aged and elderly Chinese population with prediabetes or newly diagnosed diabetes: a cross-sectional study

**DOI:** 10.1186/s12902-015-0043-0

**Published:** 2015-09-12

**Authors:** Wen Hu, Hairong Hao, Weinan Yu, Xiaojuan Wu, Hongwen Zhou

**Affiliations:** Department of Endocrinology, The First Affiliated Hospital of Nanjing Medical University, Nanjing, 210029, Jiangsu Province China; Department of Endocrinology and Metabolism, Huai’an Hospital Affiliated to Xuzhou Medical College and Huai’an Second People’s Hospital, Huai’an, 223002 China

## Abstract

**Background:**

To examine whether elevated glycosylated hemoglobin A1c (HbA1c) is associated with hyperfiltration in a middle-aged and elderly Chinese population.

**Methods:**

Anthropometric and biochemical examinations were performed in 2491 individuals from the general population, aged 40–79 years, who participated in the Huaian Diabetes Prevention Program. The estimated glomerular filtration rate (eGFR) was calculated from creatinine levels using the CKD-EPI formula. Hyperfiltration was defined as eGFR >90^th^ percentile.

**Results:**

After adjustment [for age, gender, waistline, body mass index, blood pressure, smoking, alcohol consumption, cholesterol, log(triglycerides), high-density lipoprotein, low-density lipoprotein, serum uric acid, sodium intake, hypertension, and use of angiotensin converting enzyme inhibitors or angiotensin receptor blockers], HbA1c and fasting plasma glucose (FPG) were found to be independently positively associated with eGFR. Additionally, after multivariate adjustment, the odds ratios (95 % CI) for hyperfiltration calculated for a 1-unit increase in HbA1c and FPG were 1.396 (1.089–1.790) and 1.306 (1.117–1.526), respectively. Compared with participants with HbA1c levels <5.7 %, the odds ratios (95 % CI) for hyperfiltration were 2.344 (1.025–5.364) in participants with HbA1c levels of 6.21–6.49 %, and 2.965 (1.537–5.720) in those with HbA1c levels ≥6.5 %.

**Conclusion:**

Elevated HbA1c (≥6.21 %) is associated with an increased odds of hyperfiltration in middle-aged and elderly Chinese. Longitudinal studies are needed to explore whether hyperfiltration increases the odds of diabetic nephropathy in individuals with prediabetes.

## Background

With the continuing increase in the number of patients with diabetes mellitus, diabetic nephropathy (DN) has become the most common cause of end-stage renal disease (ESRD) in China [1], the USA [2], and Europe [3]. DN develops in 40 % of patients with type 1 diabetes mellitus (T1DM) [4] and in 25 % of patients with type 2 diabetes mellitus (T2DM) [5]. Recent data from the Diabetes Control and Complications Trial-Epidemiology of Diabetes Interventions and Complications (DCCT-EDIC) study suggested that blood glucose levels at the time of the measurement of estimated glomerular filtration rate (eGFR) may bias the results [6]. Indeed, in patients with T1DM, the risk of impaired eGFR was lower for those treated early and aggressively compared with those with conventional treatments [6]. Thus, early diagnosis of DN and early intervention are very important.

Glycosylated hemoglobin (HbAlc) is widely accepted as being a good indicator of blood glucose control. In 2010, the American Diabetes Association (ADA) recommended that a HbAlc of 6.5 % or higher be used for the diagnosis of diabetes mellitus [7]. In 2012, the ADA further recommended an HbAlc of 5.7–6.4 % as a screening criterion for individuals with high risk for future diabetes (prediabetes) [8]. A community-based study has suggested that baseline HbA1c is a stronger predictor of subsequent diabetes and cardiovascular events than fasting glucose [9]. However, very few studies have focused on the relationship between an HbA1c <6.5 % and hyperfiltration.

Previous studies have indicated that patients with prediabetes (based on impaired fasting glucose [IFG], impaired glucose tolerance [IGT] or HbA1c of 5.7–6.4 %) are at high risk of future T2DM and have impaired endothelial diastolic function [10]. In addition, the IGT group showed microalbuminuria and elevated levels of urinary albumin excretion rate [10]. Measurement of eGFR and detection of microalbuminuria are the main methods recommended by the ADA, the National Kidney Foundation, and the International Society of Nephrology for the screening of DN and the monitoring of its progression in the clinical setting [11–13]. However, the UK Prospective Diabetes Study found that 51 % of patients with T2DM without albuminuria subsequently developed chronic renal insufficiency [14]. Moreover, microalbuminuria does not necessarily lead to macroalbuminuria, and in fact may regress spontaneously [15]. Furthermore, eGFR is a sensitive indicator of renal hemodynamics, and may have advantages over microalbuminuria in the detection of DN [16, 17].

Recent studies have focused on the correlation between HbA1c and the decrease in GFR in diabetes [18, 19]. However, glomerular hyperfiltration is a characteristic functional abnormality in patients with diabetes mellitus [20], and its presence is associated with an increased risk of albuminuria and DN progression [21]. The correlation between fasting plasma glucose (FPG) levels and hyperfiltration has been well established in the general population [22, 23]. However, measurement of FPG is affected by a number of factors such as sleeping, foods consumed the previous day, physical activity, sample handling, etc. [24]. Albeit not perfect, HbA1c levels are considered more stable and representative of the chronic glucose levels in an individual [25]. However, the correlation between HbA1c levels and hyperfiltration has not been definitively established in patients with diabetes; moreover, there is a paucity of data examining this correlation in individuals with prediabetes. Therefore, the present study was performed to analyze the relationship between HbA1c and eGFR in individuals without diabetes.

## Methods

### Ethics statement

This cross-sectional study was part of the Huaian Diabetes Prevention Program (ChiCTR-TRC-14005029) and was approved by the Huaian Second Hospital Ethics Committee, XuZhou Medical University, China. Written informed consent was obtained from all participants in this study.

### Study population

In the present study, 5431 subjects (aged 40–79 years) attending annual routine health examinations under the auspices of the local governments between August and December 2014 at the health examination center of Huaian Second Hospital, Affiliated Hospital of Xuzhou Medical College in Huaian (Jiangsu, China) were enrolled. Subjects were subsequently excluded from the analysis if any of the following criteria applied: (1) data were missing for calculation of the eGFR (*n* = 358); (2) previously diagnosed diabetes mellitus (*n* = 528); (3) previously diagnosed renal disease, including autoimmune or drug-induced kidney disease, nephritis, renal fibrosis or renal failure, or previous kidney transplant with ongoing renal dialysis (*n* = 146); (4) previously diagnosed hepatic disease including fatty liver, liver cirrhosis and autoimmune hepatitis (*n* = 1132); (5) peripheral artery disease (*n* = 108); (6) coronary heart disease (CHD) including myocardial infarction and angina pectoris (*n* = 479); (7) any malignant disease (*n* = 8); or (8) eGFR <60 mL/min/1.73 m^2^ (*n* = 181). Therefore, 2491 subjects (1624 women) were deemed eligible for the analysis.

### Data collection

Demographic characteristics, lifestyle information, and medical history were obtained by trained investigators using a standard questionnaire. Body mass index (BMI) was calculated as weight (kg) divided by height squared (m^2^). Blood pressure (BP) was measured three consecutive times (HEM-752 Fuzzy; Omron Company, Dalian, China), and the mean value was used in the analysis. Venous blood samples were collected between 07:00 and 09:00, after overnight fasting, for the measurement of FPG, creatinine (CREA), total cholesterol (TC), triglycerides (TG), low-density lipoprotein cholesterol (LDL-c), and high-density lipoprotein cholesterol (HDL-c). HbA1c was measured by high performance liquid chromatography (Variant II and D-10 Systems, Bio-Rad Laboratories Inc., Hercules, CA, USA). All quality control criteria of the ADA were applied for HbA1c measurement [8], and the laboratory was properly qualified.

Diabetes was defined according to the 2012 ADA criteria [8]: FPG ≥126 mg/dl (7.0 mmol/L); or 2-h plasma glucose in the 75-g oral glucose tolerance test (OGTT) ≥200 mg/dL (11.1 mmol/L); and/or HbA1c ≥6.5 %. Prediabetes was also defined according to the 2012 ADA criteria [8]: 100 mg/dL (5.6 mmol/L) ≤ FPG ≤ 125 mg/dL (6.9 mmol/L) (IFG); or 140 mg/dL (7.8 mmol/L) ≤2-h plasma glucose in the 75-g OGTT ≤199 mg/dL (11.0 mmol/L) (IGT); or HbA1c 5.7-6.4 %. Normal glucose metabolism (NGM) was defined as HbA1c <5.6 %, FPG <100 mg/dL (5.6 mmol) and 2-h plasma glucose in the 75-g OGTT <140 mg/dL (7.8 mmol/L). Based on these criteria, 213 of the 2491 subjects included in the analysis were considered to have newly diagnosed type 2 diabetes mellitus (T2NDM), 1037 were considered to have prediabetes, and 1241 were considered to have NGM.

eGFR was calculated from creatinine levels using the CKD-EPI formula [26]. Normal eGFR was defined as ≥90 mL/min/1.73 m^2^; renal hyperfiltration as an absolute GFR >102 mL/min/1.73 m^2^ (90^th^ percentile in all subjects); and mildly reduced eGFR as 60–90 mL/min/1.73 m^2^.

### Statistical analysis

The continuous variables in this study exhibited normal or approximately normal distributions, and are presented as means ± standard deviations (SDs). Categorical variables are presented as numbers (%). Comparisons between groups were made using the Student’s t test for continuous data and the chi-square test for categorical data.

After verifying the assumption of a linear relationship between the dependent and independent variables that were introduced into a linear regression model (assessed using a histogram of the residuals, together with a scatter plot of the standardized residuals to the standardized predicted values in different models, as described below), multivariate linear regression analysis was used to estimate the association of glucose metabolism type with eGFR. Identification of patients with hyperfiltration was done by selecting all participants >90^th^ percentile in the distribution of residuals from a multivariate linear regression analysis where we used absolute GFR values as the dependent variable and sex, age, weight, height, and use of ACE inhibitors or ARB as the independent adjusting variables. Then, 249 subjects (147 women) were defined as being with hyperfiltration, for a mean GFR of 102.27 (range 90.7–135.7) mL/min/1.73 m^2^.

Three models were constructed for each component of glucose metabolism. The first model was not adjusted. The second model was adjusted for age, gender, waistline, BMI, systolic blood pressure, and diastolic blood pressure. The third model was adjusted for age, gender, waistline, BMI, systolic BP, diastolic BP, cholesterol, log(triglyceride), HDL-c, LDL-c, serum uric acid, alcohol consumption, smoking status, sodium intake (6 g/day), the presence of hypertension (%), and the use of angiotensin converting enzyme inhibitors (ACEIs) or angiotensin receptor blockers (ARBs).

For analysis, the subjects were divided into five groups based on stratification of glucose metabolism levels using the 50^th^, 75^th^, 90^th^ and 95^th^ percentiles as cut-off points. For HbA1c, the groups were: Q1, HbA1c <5.70 %; Q2, HbA1c 5.70–6.00 %; Q3, HbA1c 6.01–6.20 %; Q4, HbA1c 6.21–6.49 %; and Q5, HbA1c ≥6.5 %. For FPG, the groups were: Q1, FPG <5.40 mmol/L; Q2, FPG 5.40–5.70 mmol/L; Q3, FPG 5.71–6.30 mmol/L; Q4, FPG 6.31–6.99 mmol/L; and Q5, FPG ≥7.0 mmol/L). The associations of glucose metabolism (with FPG and HbA1c quintiles introduced as dummy ordinal independent variables) with the odds of hyperfiltration (as defined above) were estimated using multivariate logistic regression analysis in the same three models described above. *P*-values for the trends were calculated by Spearman correlation analysis of categorical variables and odds ratios (ORs) for the different groups, scored 0, 1, 2 and 3, respectively. *P* < 0.05 was considered statistically significant. All statistical analyses were performed using SPSS 16.0 (SPSS Inc., Chicago, IL, USA).

## Results

### Characteristics of the study participants

The 2491 subjects (1624 women) included in the study were divided into three groups (NGM, prediabetes, and T2NDM). As shown in Table 1, age, BMI, FPG, HbA1c, TC, TG, LDL-c, SUA, alcohol consumption, sodium intake (>6 g/day), and the proportion of subjects with hypertension were significantly higher in the prediabetes and T2NDM groups than in the NGM group (*P* < 0.05), while HDL-c levels were significantly lower (*P* = 0.003). Interestingly, eGFR and the proportion of subjects with hyperfiltration were lower in the prediabetes group but higher in the T2NDM group. There was no significant difference between the three groups in systolic BP (SBP), diastolic BP (DBP), blood urea nitrogen (BUN), CREA, gender, and smoking status.

**Table 1:** Characteristics of the study participants

	NGM (*n* = 1241)	Prediabetes (*n* = 1037)	T2NDM (*n* = 213)	*P* value
Male (%)	34.6	34.0	39.0	0.386
Age (years)	59.36 ± 7.39	61.04 ± 7.43	63.08 ± 7.34	<0.001
Smoking (%)	16.3	17.7	18.8	0.577
Consumption of alcohol (%)	15.4	14.9	17.9	0.033
Excess sodium intake (>6 g/d) (%)	14.4	15.7	20.7	<0.001
Hypertension (%)	39.7	43.5	47.9	0.033
Use of ACEIs or ARBs (%)	5.3	4.4	3.7	0.514
Waistline (cm)	82.26 ± 8.88	83.14 ± 9.31	85.43 ± 11.97	<0.001
BMI (kg/m^2^)	23.90 ± 2.96	24.32 ± 3.21	25.00 ± 3.82	<0.001
SBP (mmHg)	140.46 ± 20.11	140.42 ± 38.31	140.86 ± 20.30	0.597
DBP (mmHg)	84.75 ± 13.94	84.31 ± 14.41	83.05 ± 15.73	0.573
TC (mmol/L)	5.12 ± 0.85	5.25 ± 0.85	5.33 ± 0.91	<0.001
TG (mmol/L)	1.61 (0.5, 12.7)	1.67 (0.3, 18.1)	1.85 (0.8, 19.8)	<0.001
HDL-c (mmol/L)	1.45 ± 0.61	1.39 ± 0.47	1.36 ± 0.56	0.003
LDL-c (mmol/L)	2.68 ± 0.68	2.80 ± 0.70	2.88 ± 0.75	<0.001
SUA (mg/dL)	4.76 ± 1.22	4.80 ± 1.33	4.90 ± 1.39	0.018
BUN (mmol/L)	5.18 ± 1.29	5.10 ± 1.25	5.02 ± 1.33	0.076
CREA (mmol/L)	0.78 ± 0.15	0.79 ± 0.15	0.78 ± 0.16	0.650
eGFR (mL/min per 1.73 m^2^)	89.39 ± 11.76	88.31 ± 11.65	88.76 ± 12.83	0.048
eGFR stage				0.013
>102 (%)	13.7	9.5	14.1	
90-102 (%)	39.2	39.4	39.4	
60-90 (%)	47.1	50.2	46.5	
HbA1c (%)	5.32 ± 0.27	5.92 ± 0.18	7.13 ± 1.05	<0.001
FPG (mg/dL)	94.86 ± 10.08	98.18 ± 9.23	131.76 ± 15.30	<0.001

Data are presented as the mean ± SD, number (as %), or median (range), as appropriate. Comparisons between the three groups were made using ANOVA or Fisher’s exact test, as appropriate

International system of units (SI) conversion: plasma glucose, 1 mg/dL = 1/18 mmol/L; serum uric acid, 1 mg/dL = 59.5 mmol/L; serum creatinine, 1 mg/dL = 88.41 μmol/L

*ACEI* angiotensin converting enzyme inhibitor; *ARB* angiotensin receptor blocker; *BMI* body mass index; *BUN* blood urea nitrogen; *CREA* serum creatinine; *DBP* diastolic blood pressure; *e-GFR* estimated glomerular filtration rate; *FPG* fasting plasma glucose; *HDL-c* high-density lipoprotein cholesterol; *LDL-c* low-density lipoprotein cholesterol; *T2NDM* newly diagnosed type 2 diabetes mellitus; *NGM* normal glucose metabolism; *SBP* systolic blood pressure; *SUA* serum uric acid; *TC* total cholesterol; *TG* triglycerides

Among all subjects, 249 subjects (147 women) were defined as being with hyperfiltration, for a mean GFR of 102.27 (range 90.7–135.7) mL/min/1.73 m^2^.

### Multiple linear regression analysis

As presented in Table 2, an approximately linear relationship was found, particularly in models 2 and 3. In model 1, the HbA1c and FPG levels were independently negatively related to eGFR. However, after adjustment for age, gender, waistline, BMI, SBP, DBP, cholesterol, log(triglyceride), LDL-c, HDL-c, alcohol consumption, smoking status, hypertension, and the use of ACEIs or ARBs in models 2 and 3, HbA1c and FPG levels were found to be positively related to eGFR in participants with prediabetes and those with T2NDM.

**Table 2:** Multiple linear regression analysis of the relationship between HbA1c and eGFR

	Fasting plasma glucose (per mmol/L)			HbA1c (per % unit)		
	b Coefficient	95 % CI	*P* value	b Coefficient	95 % CI	*P* value
Model 1	−0.466	(−1.037, −0.087)	0.015	−0.471	(−1.363, 0.078)	0.203
Model 2	0.424	(−0.016, 0.864)	0.059	1.044	(0.378, 1.711)	0.002
Model 3	0.761	(0.340, 1.180)	<0.001	1.259	(0.625, 1.892)	<0.001

Model 1: not adjusted; Model 2: adjusted for age, gender, waistline, body mass index, systolic blood pressure and diastolic blood pressure; Model 3: Model 2 plus adjustment for cholesterol, Log (triglyceride), high-density lipoprotein cholesterol, low-density lipoprotein cholesterol, serum uric acid, alcohol consumption, smoking status, sodium intake (6 g/day), hypertension, and use of angiotensin converting enzyme inhibitors or angiotensin receptor blockers. 95 % CI, 95 % confidence interval; eGFR, estimated glomerular filtration rate; HbA1c, glycated hemoglobin

### Multiple logistic regression analysis

As shown in Table 3, the association between increased HbA1c levels and increased odds of hyperfiltration was analyzed in the three models. In model 1, both the HbA1c and FPG levels were negatively related to GFR, but after adjustment for age, gender, waistline, BMI, SBP and DBP (model 2) or age, gender, waistline, BMI, SBP, DBP, TC, log(triglyceride), LDL-c, HDL-c, alcohol consumption, smoking status, hypertension, and the use of ACEIs or ARBs (model 3), HbA1c and FPG levels showed a positive linear relationship with an increased odds of hyperfiltration, with the highest quintile groups (FPG ≥7.0 mmol/L [126 mg/dL] and HbA1c ≥6.5 %) showing significantly increased odds. In model 3 (after multivariate adjustment), the OR (95 % CI) for hyperfiltration (relative to quintile Q1, HbA1c <5.7 %) was 2.344 (1.025–5.364) in subjects with HbA1c levels of 6.21–6.49 % (Q4), and 2.965 (1.537–5.720) in those with HbA1c levels >6.5 % (Q5) (both *P* < 0.05).

**Table 3:** Multiple logistic regression analyses of odds ratios for hyperfiltration

	Fasting plasma glucose (per mmol/L)				HbA1c (per % unit)			
		OR	95 % CI	*P* value		OR	95 % CI	*P* value
Model 1	Fasting glucose, per mmol/L	0.947	(0.826-1.086)	0.434	HbA1c, per % unit	0.850	(0.689-1.048)	0.128
	Q1 (<5.40)	1			Q1 (<5.70)	1		
	Q2 (5.40-5.70)	0.654	(0.482-0.887)	0.006	Q2 (5.70-6.00)	0.644	(0.443-0.936)	0.021
	Q3 (5.71-6.30)	0.786	(0.499-0.919)	0.012	Q3 (6.01-6.20)	0.593	(0.405-0.870)	0.007
	Q4 (6.31-6.99)	0.692	(0.338-0.994)	0.047	Q4 (6.21-6.49)	1.234	(0.992-2.719)	0.053
	Q5 (≥7.0)	0.871	(0.689-1.698)	0.734	Q5 (≥6.5)	0.900	(0.583-1.390)	0.819
Model 2	Fasting glucose, per mmol/L	1.222	(1.064-1.405)	0.005	HbA1c, per % unit	1.301	(1.027-1.648)	0.029
	Q1 (<5.40)	1			Q1 (<5.70)	1		
	Q2 (5.40-5.70)	0.820	(0.545-1.234)	0.341	Q2 (5.70-6.00)	0.779	(0.522-1.222)	0.300
	Q3 (5.71-6.30)	0.884	(0.572-1.367)	0.579	Q3 (6.01-6.20)	0.876	(0.564-1.361)	0.557
	Q4 (6.31-6.99)	1.181	(0.545-1.2.563)	0.673	Q4 (6.21-6.49)	2.497	(1.144-5.440)	0.025
	Q5 (≥7.0)	3.719	(1.969-7.024)	<0.001	Q5 (≥6.5)	2.424	(1.315-4.469)	0.005
Model 3	Fasting glucose, per mmol/L	1.306	(1.117-1.526)	0.001	HbA1c, per % unit	1.396	(1.089-1.790)	0.008
	Q1 (<5.40)	1			Q1 (<5.70)	1		
	Q2 (5.40-5.70)	0.845	(0.547-1.305)	0.448	Q2 (5.70-6.00)	0.968	(0.623-1.504)	0.885
	Q3 (5.71-6.30)	1.144	(0.731-1.791)	0.557	Q3 (6.01-6.20)	0.945	(0.598-1.496)	0.811
	Q4 (6.31-6.99)	2.369	(1.255-4.473)	0.097	Q4 (6.21-6.49)	2.344	(1.025-5.364)	0.044
	Q5 (≥7.0)	4.889	(2.454-9.739)	<0.001	Q5 (≥6.5)	2.965	(1.537-5.720)	<0.001

Model 1: not adjusted; Model 2: adjusted for age, gender, waistline, body mass index, systolic blood pressure and diastolic blood pressure; Model 3: Model 2 plus adjustment for cholesterol, Log (triglyceride), high-density lipoprotein cholesterol, low-density lipoprotein cholesterol, serum uric acid, alcohol consumption, smoking status, sodium, hypertension, and use of angiotensin converting enzyme inhibitors or angiotensin receptor blockers. 95 % CI, 95 % confidence interval; HbA1c, glycated hemoglobin; OR, odds ratio

## Discussion

The present study has found that in a Chinese middle-aged and elderly population without a history of diabetes, HbA1c was positively associated with hyperfiltration independently of age, sex, BMI, waistline, BP, smoking status, alcohol consumption, hypertension status, and TC, TG, LDL-c and HDL-c levels. A similar association was found between FPG and hyperfiltration. However, since the two conditions often coexist, the results do not exclude the association of both acutely and chronically elevated glucose levels with eGFR, but cannot confirm an independent association. Furthermore, after multivariate adjustment, it was determined that the odds of hyperfiltration were significantly increased in patients with HbA1c ≥6.21 % and FPG ≥7.0 mmol/L. Subjects with HbA1c levels of 6.21–6.49 % had a 2.34-times higher odds of developing hyperfiltration than those with HbA1c <5.7 %. This suggests that hyperfiltration occurs not only in newly developed diabetes but also in prediabetes. Thus, more attention should be paid to subjects with HbA1c levels ≥6.21 % in order to maximize the prevention of hyperfiltration in patients without overt diabetes.

Some studies, but not all, have shown an association between hyperfiltration and the subsequent development of nephropathy in subjects with diabetes [27]. It is important to clarify the relationship between blood glucose and hyperfiltration to allow the early diagnosis of renal damage. GFR is increased significantly in dogs by a continuous glucose infusion for 6 days, which produces a modest rise in serum glucose from 6.5 to 7.1 mmol/L [28]. Consistent with the present study, experimental studies in healthy subjects have shown that GFR is increased by an acute glucose infusion (plasma glucose to 7.0 mmol/L) [29]. Recent research into hyperfiltration has focused on the relationship between an acute blood glucose rise and eGFR, rather than the effects of a chronic elevation in blood glucose level. A previous study of 1560 Norwegians, aged 50–62 years, without diabetes, has assessed the association between elevated HbA1c levels and GFR [22]. In the Norwegian investigation, elevated HbA1c and borderline hyperglycemia (FPG >5.6 mmol/L) were found to be associated with hyperfiltration; however, the cut-off for FPG differed from that used in the present study, and they analyzed HbA1c as a continuous variable. Consistent with our findings, Okada et al. found that hyperfiltration was associated with HbA1c levels in a cohort of Japanese subjects with prediabetes [30]. In addition, Hou et al. have reported that greater fluctuations between FPG and 2-h postload glucose were associated with higher odds of hyperfiltration [31].

The present study differs from previous investigations of hyperfiltration [29, 22, 32]. First, subjects for this cross-sectional study were selected from a Chinese population free of CHD, diabetes, peripheral arterial disease, or chronic kidney disease, which shows a lower GFR and lower rate of GFR decrease than Western populations. Second, the definition of hyperfiltration that was used differed from that of previous studies [33, 34]. The clinical relevance of hyperfiltration is based on a proposed pathological effect of increased single nephron GFR, which cannot be measured in humans. Therefore, we selected an absolute GFR above the 90^th^ percentile as the definition of hyperfiltration, rather than an arbitrary threshold that has ranged in various studies from 125 to 140 mL/min/1.73 m^2^. Third, extensive covariate data, including age, gender, BMI, cigarette smoking status, alcohol consumption, sodium intake, hypertension history, SBP, DBP, log(TG), TC, HDL-c, LDL-c, FPG, SUA, eGFR, and waistline were used in the statistical analysis. The present study is supported by two previous ones [22, 30], but these two studies were performed in different populations and using different definitions of hyperglycemia and high HbA1c levels. In addition, they mainly focused on FPG, which are biased by a number of factors [24]. Taken together, these results may help clinicians to intervene earlier to prevent kidney damage due to hyperfiltration.

Creatinine-based equations for estimating GFR include the Cockcroft-Gault equation proposed in 1976 [35], the Modification of Diet in Renal Disease (MDRD) study equation proposed in 1999 [36], and the CKD-EPI equation proposed in 2009 [15]. The Cockcroft-Gault equation has now been supplanted by the MDRD study equation and the CKD-EPI equation [37]. A recent study performed in South Asians, aged 40 years or older (as in the present study) demonstrated that the CKD-EPI equation was more accurate and precise in estimating GFR than the MDRD study equation [38]. Therefore, the CKD-EPI equation was selected for the calculation of eGFR in the present study.

The etiology of increased GFR in the setting of elevated blood glucose and HbA1c is incompletely understood, but has been attributed to the effect of hyperglycemia on the renin-angiotensin-aldosterone system (RAAS). Miller et al. demonstrated that hyperfiltration responses to clamped hyperglycemia are related to intrarenal RAAS activation [39]. Moreover, proximal sodium reabsorption is higher in individuals with IFG than in subjects with normal FPG [32]. To the best of our knowledge, there is no current evidence for a direct effect of glucose on creatinine, suggesting that the positive relationship between blood glucose and eGFR is likely reflective of changes in GFR.

Of course, the present study has some limitations. First, the study sample might not be completely representative of the general population. Second, the methods used to measure eGFR may be influenced by non-GFR factors such as body composition and glycemic status [38], and by non-traditional factors such as asymmetric dimethylarginine (ADMA), symmetric dimethylarginine, L-arginine/ADMA ratio, and insulin resistance [40]. In addition, the mean eGFR of the Chinese subjects in our study was lower than that of Western populations [29, 22, 32], which may be due to a number of lifestyle and genetic factors. Third, a cross-sectional study cannot infer causality between HbA1c, hyperfiltration, and nephropathy. Therefore, longitudinal studies are needed to investigate whether the hyperfiltration associated with increased HbA1c is a risk factor for renal injury in the general population. Fourth, this study did not include consideration of insulin resistance, a factor that some have suggested may affect hyperfiltration [22, 41], but which is still controversial [42]. Finally, prediabetes was defined based on FPG and HbA1c only; OGTT was not performed, and it is possible that some patients may have been missed [43].

## Conclusions

Elevated HbA1c levels are associated with increased odds of hyperfiltration in a middle-aged and elderly Chinese population. It is possible that hyperfiltration associated with elevated HbA1c (≥6.21 %) may be one of several mechanisms that cause renal injury in this population of patients.

## Abbreviations

HbA1c: Hemoglobin A1c
eGFR: Estimated glomerular filtration rate
FPG: Fasting plasma glucose
DN: Diabetic nephropathy
ESRD: End-stage renal disease
T2DM: Type 2 diabetes mellitus
DCCT-EDIC: Diabetes control and complications trial-epidemiology of diabetes interventions and complications
ADA: American Diabetes Association
IFG: Impaired fasting glucose
IGT: Impaired glucose tolerance
CHD: Coronary heart disease
BMI: Body mass index
BP: Blood pressure
TC: Total cholesterol
TG: Triglycerides
LDL-c: Low-density lipoprotein cholesterol
HDL-c: High-density lipoprotein cholesterol
OGTT: Oral glucose tolerance test
NHM: Normal glucose metabolism
SDs: Standard deviations
ACEIs: Angiotensin converting enzyme inhibitors
ARBs: Angiotensin receptor blockers
MDRD: Modification of diet in renal disease
RAAS: Renin-angiotensin-aldosterone system
